# Separase activity distribution can be a marker of major molecular response and proliferation of CD34^+^ cells in TKI-treated chronic myeloid leukemia patients

**DOI:** 10.1007/s00277-020-04007-4

**Published:** 2020-04-06

**Authors:** Birgit Spiess, Helga Kleiner, Johanna Flach, Alice Fabarius, Susanne Saussele, Wolf-Karsten Hofmann, Wolfgang Seifarth

**Affiliations:** 1 Department of Hematology and Oncology, University Hospital Mannheim, Medical Faculty Mannheim, Heidelberg University, Mannheim, Germany; 2 Hämatologie und Onkologie, III. Medizinische Klinik, Wissenschaftliches Labor, Universitätsklinikum Mannheim GmbH, Pettenkoferstraße 22, 68169 Mannheim, Germany

**Keywords:** *ESPL1*/separase, Chronic myeloid leukemia (CML), *BCR-ABL1* expression, Major molecular remission (MMR), Leukemic stem cell (LSC), Leukemic niche

## Abstract

Separase, a cysteine endopeptidase, is a key player in mitotic sister chromatid separation, replication fork dynamics, and DNA repair. Aberrant expression and/or altered separase proteolytic activity are associated with aneuploidy, tumorigenesis, and disease progression. Since genomic instability and clonal evolution are hallmarks of progressing chronic myeloid leukemia (CML), we have comparatively examined separase proteolytic activity in TKI-treated chronic phase CML. Separase proteolytic activity was analyzed on single cell level in 88 clinical samples and in 14 healthy controls by a flow cytometric assay. In parallel, *BCR-ABL1* gene expression and replication fork velocity were measured by qRT-PCR and DNA fiber assays, respectively. The separase activity distribution (SAD) value indicating the occurrence of MNCs with elevated separase proteolytic activity within samples was found to positively correlate with *BCR-ABL1* gene expression levels and loss of MMR (relapse) throughout routine *BCR-ABL1* monitoring. Analyses of CD34^+^ cells and MNCs fractionized by flow cytometric cell sorting according to their separase activity levels (H- and L-fractions) revealed that CD34^+^ cells with elevated separase activity levels (H-fractions) displayed enhanced proliferation/viability when compared with cells with regular (L-fraction) separase activity (mean 3.3-fold, *p* = 0.0011). *BCR-ABL1* gene expression positivity prevailed in MNC H-fractions over L-fractions (42% vs. 8%, respectively). Moreover, expanding CD34^+^ cells of H-fractions showed decreased replication fork velocity compared with cells of L-fractions (*p* < 0.0001). Our data suggests an association between high separase activity, residual *BCR-ABL1* gene expression, and enhanced proliferative capacity in hematopoietic cells within the leukemic niche of TKI-treated chronic phase CML.

**Electronic supplementary material:**

The online version of this article (10.1007/s00277-020-04007-4) contains supplementary material, which is available to authorized users.

## Introduction

Improved therapy regimen employing first-, second-, and third-generation tyrosine kinase inhibitors (TKI) directed at the abnormal *BCR-ABL1* fusion tyrosine kinase (TK) lead to achievements of durable cytogenetic (CyR) and molecular remissions (MR) in patients with chronic myeloid leukemia (CML). The survival rate of the majority of patients is approaching that of the general population [1–3]. For patients that have achieved a permanent deep MR under TKI treatment, the conception of treatment-free remission (TFR) has been supported. Despite deep MR achievement about 40–60% of patients display increase in *BCR-ABL1* transcript levels and need treatment reconstitution. Only about half of all patients are able to have sustained TFR [4]. It seems that despite significant decreases in *BCR-ABL1* mRNA levels under TKI long-term therapy, the persistence of residual CML clones with low *BCR-ABL1* expression and insensitivity to TKI treatment in the bone marrow (BM) compartment makes disease eradication by TKI treatment alone unlikely [5, 6]. Recent evidence suggests that kinase activity of the BCR-ABL1 oncoprotein in CML stem cells is inhibited by TKI treatment without affecting CML stem cell survival [7, 8]. Obviously, additional cellular mechanisms promote CML stem cell survival and maintenance, rendering these cells TKI-resistant and eventually promote molecular relapse [9, 10].

Since only few factors for leukemic stem cell (LSC) dormance are identified so far, it is important to explore new targets and to develop potent small molecules for eradication of the leukemia clone [11–13].

*ESPL1*/separase, a cysteine endopeptidase, is a key player of chromosomal segregation. In mitotic anaphase, it accomplishes proteolytic cleavage of cohesin, a “glue” multi-protein complex that holds sister-chromatids together [14–16]. Proper temporal and spatial activation of separase proteolytic activity warrants chromosomal fidelity by unleashing sister-chromatids at anaphase onset and establishing an accurate chromosomal segregation [17]. Furthermore, separase is an essential prerequisite for semiconservative centriole duplication as disengagement of mother and daughter centrioles by proteolysis licenses the cell cycle–associated duplication of centrosomes [18]. Separase on interphase chromosomes has been reported to be involved in controlling replication fork speed and in postreplicative DNA double-strand break repair (DSBR). There, separase acts locally to cleave distinct cohesin molecules and to facilitate homology-directed repair (HDR) thereby preventing oncogenic transformation [19–22]. Due to these interphase activities, separase has been recently proposed to be active throughout the cell cycle on a low level and there may be a mere increase in separase activity during metaphase contrary to the common believes that separase is inactive throughout cell cycle except for metaphase/anaphase [23].

In human cancers, *ESPL1*/separase is frequently overexpressed and the resulting deregulated proteolytic activity is associated with premature segregation of chromatids and/or formation of anaphase bridges from lagging chromosomes [24]. Moreover, unscheduled (cell cycle uncoupled) activation of separase can lead to aberrant high numbers of centrosomes (i.e., centrosome amplification) and subsequently to a defective mitotic spindle apparatus [25]. Both defects cause the emergence of aberrant karyotypes (aneuploidy), a hallmark of most advanced human malignancies [24, 26–30]. Overexpression of separase in the mammary gland of a MMTV-*ESPL1* mouse model led to the development of highly aneuploid mammary carcinomas with high levels of chromosomal instability and aggressive disease phenotypes [31]. Consequently, separase has been identified as an aneuploidy promoter that, when overexpressed and hyperactive, functions as an oncogene and renders cells susceptible not only for chromosomal missegregation-induced aneuploidy but also for DNA damage and loss of key tumor suppressor gene loci associated with tumorigenesis and disease progression [31–33].

In search for molecular mechanisms that contribute to the survival of LSC and clonal evolution during TKI-related dormance, we set out to investigate primary cells with elevated separase activity levels derived from the peripheral blood of 88 CML patients. We show that the occurrence of these cells in diagnostic samples can be a marker for loss of major molecular response (MMR) and concurs with *BCR-ABL1* gene expression positivity. Furthermore, primary CD34^+^ cells with elevated separase activity levels feature increased proliferation capacity in vitro and show decreased replication fork velocity in DNA fiber assays. The potential impact of these findings for clonal evolution and disease progression as indicated by loss of MMR and dormance of the malignant clone within the leukemic niche of TKI-treated CML in terms of TKI stopping trials is discussed.

## Methods

### Patients and control samples

In general, clinical sample acquisition was based solely on the availability of a sufficient number of CD34^+^ cells irrespective of longitudinal treatment journey, TKI treatment regimen, or response criteria such as time to relapse. For determination of the separase activity distribution (SAD) values from mononuclear cells (MNCs) by separase activity cell sorting (Fig. 3a), 88 peripheral blood (PB) samples of 88 CML patients in chronic phase under TKI treatment were analyzed in total and grouped into two cohorts according to their clinical status. The first cohort comprised 41 CML patients (20 female, 21 male, median age 55 years, range 22–80 years) who were classified as “no major molecular remission” (noMMR). The second cohort comprised 47 CML patients (20 female, 27 male, median age 60 years, range 26–90 years) classified as “MMR and deep MR” including patients with molecular response (MR) to TKI treatment with 4-log (MR_4_), 4.5-log (MR_4.5_), and 5-log (MR_5_) reduction in *BCR-ABL1* transcript levels according to the international standard (IS, [34]. Blood sampling was performed in the context of regular therapeutic monitoring. MNCs of healthy donors (*n* = 14, 9 female, 5 male, median age 43 years, range 18–88 years) served as non-leukemic controls. Proliferation and viability assays from CD34^+^ cells after 3 days of cultivation were performed using a subset of 23 PB and/or BM samples of 22/88 CML patients (6 female, 16 male, median age 60 years, range 18–82 years) (Fig. 4c). *BCR-ABL1* expression in MNCs was measured by quantitative reverse transcriptase polymerase chain reaction (qRT-PCR) from 48 PB and/or BM samples of 42/88 CML patients (12 female, 30 male, median age 57 years, range 18–78 years) (Fig. 4d).

The study has been approved by the institutional ethics committee (Medizinische Ethikkommission II der Medizinischen Fakultät Mannheim der Ruprecht Karls-Universität Heidelberg, no. 2013-509N-MA). All patients included in the study provided written informed consent in accordance with the Declaration of Helsinki.

### Clinical sample preparation

Clinical samples were processed by ficollization according to manufacturer’s instructions (Ficoll-Paque™ PREMIUM no. 17-5442-03; GE Healthcare Bio-Sciences AB, Uppsala, Sweden). Using ficollization, the MNCs and CD34^+^ cells were separated from the clinical samples as different cell compounds. Separation of the CD34^+^ cells was realized using an anti CD34-APC-antibody-based separation (APC-Mouse Anti Human CD34 no. 55824; BD Biosciences, Heidelberg, Germany) during fluorescence-activated cell sorting (FACS). MNCs were solely prepared from PB samples, CD34^+^ cells from both, and PB and BM samples.

### Measurement of separase proteolytic activity and cell sorting for MNCs

The flow cytometric separase activity assay was performed as previously described [35]. In brief, the flow cytometry–based assay utilizes a Cy5- and rhodamine 110 (Rh110)-biconjugated Rad21 cleavage site peptide ([Cy5-D-R-E-I-M-R]2-Rh110) as smart probe and intracellular substrate for detection of separase enzyme activity in living cells. As measured by Cy5 fluorescence in pilot experiments, the cellular influx/uptake of the fluorogenic peptide was fast and reached saturation within 60 min and 210 min of incubation in human MNCs and in human histiocytic lymphoma U937 cells, respectively. Separase activity was recorded as the intensity of Rh110 fluorescence released after intracellular peptide cleavage providing a linear signal gain within a 90- to 180-min time slot. Compared with conventional cell extract–based methods, the flow cytometric assay delivers equivalent results but is more reliable and bypasses the problem of vague loading controls for normalization and of unspecific proteolysis associated with whole cell extracts. Especially suited for the investigation of blood- and bone marrow–derived hematopoietic cells, the flow cytometric separase assay allows generation of separase activity profiles that tell about the number of separase-positive cells within a sample, i.e., cells that currently progress through mitosis and about the range of intercellular variation in separase activity levels within a cell population. In this survey, a monoconjugated reporter peptide (Ac-D-R-E-I-M-R]2-Rh110) was used (10 μM final concentration, 90-min incubation) for measurement of separase proteolytic activity in MNCs and CD34^+^ hematopoietic progenitor cell preparations since the Cy5 channel was used for simultaneous CD34^+^ cell sorting. Fluorescence (10,000 signaling events in the viable population) of rhodamine 110 (Rh110) (Ex_max 488 nm_, Em_max 535 nm_) was measured by FACS using a flow cytometer FACSAria (Beckton Dickinson, San José, USA) or FACSMelody (Beckton Dickinson, San José, USA). Discrimination of background fluorescence, cellular debris, cell doublets, and gating of cells displaying separase activity (positive for Rh110) was performed by Kaluza software (version 1.3, Beckman Coulter, Inc., Krefeld, Germany). Forward scatter (FSC) files were imported in Microsoft Excel 2010 (Microsoft, Redmond, WA, USA) for further analysis.

Unstained cells (without peptide) were used as negative controls. The separase activity distribution (SAD) value was calculated as quotient of the mean RFU (relative fluorescence units) of 0.5% separase-positive cells above the 99.5 percentile divided by mean RFU of 99.5% of separase-positive cells below the 99.5 percentile (SAD value = *M*_0.5_/*M*_99.5_). Thus, the SAD value serves as a numerical value of intercellular separase activity distribution among cells in the sample of interest. While measuring separase activity, the MNCs were sorted in H- and L-fractions and resuspended in 200 μl lysis buffer (from μMACS One-step cDNA kit, Miltenyi Biotech, Bergisch Gladbach, Germany) for consecutive *BCR-ABL1* gene expression testing. Gating strategy for sorting separase-active cells into fractions with high (H-fraction) and low (regular, L-fraction): separase proteolytic activity cutoffs were established empirically based on the borders of noMMR patients between 0.5 and 99.5 percentiles as depicted in Fig. 2d. Therefore, no H-fractions with considerable number of cells for healthy and MMR groups were achieved.

During establishment of our separase assay, we have used dual-labeled reporter peptide for testing the kinetics of uptake/influx of reporter peptide (Cy5- and rhodamine 110 (Rh110)-biconjugated Rad21 cleavage site peptide ([Cy5-D-R-E-I-M-R]2-Rh110) as smart probe) and efflux of the released label (Rh110) under experimental conditions within various cell lines and cell types (leukemic cell lines, MNCs, CD34^+^ progenitors). We found and stated in [35] that irrespective to the cell type at 10 μM concentration, the uptake of the reporter peptide is very fast and all cells are Cy5-positive after 1 min of incubation reaching saturation with the peptide after 210 min of incubation as the uptake/influx follows the principle of a steady-state equilibrium. For peripheral blood MNCs, we observed reporter peptide uptake equilibrium after 60 min of incubation (compare Online Resource 1), suggesting that the concentration of uncleaved reporter peptide is always available in molar excess and not limiting the cleavage. The continuous influx/diffusion of fresh reporter peptide keeps the balance between outside and inside. When separase is active within a cell, a certain amount of peptidic substrate per minute is cleaved and the Rh110 as fluorescent label is released accumulating in the cell.

Moreover, as released RH110 fluorophore accumulates within the cells, the Rh110 fluorophore itself starts to diffuse out of the cell. The higher the intracellular Rh110 concentration compared with the environmental medium, the faster is the undesired efflux. Therefore, the measured separase activity (Rh110 fluorescence) is the result of the balance between continuous cleavage and undesired efflux and a function of the incubation time (until it reaches a steady-state level). It resembles a perforated bucket, always losing some of its content resulting in a distinct level of content (= equilibrium). As a matter of fact, after 90 min of incubation, the prevailing equilibrium on single cell level is measured by flow cytometry. This contrasts with regular flow cytometric analyses where antibodies are used for staining of distinct numbers of fixed target molecules. There, in the sense of an end point reaction, label and signal will accumulate only as long as free targets are available and an intracellular reference signal for normalization may be necessary for quantification because signals may correlate with incubation time and target and antibody ratio. Not so in our separase activity assay. Provided that reporter peptide is always available in molar excess, we have shown that the mentioned signal equilibrium is constant in MNCs between 60 and 120 min of incubation, making internal normalization dispensable (at 90 min of standard incubation time). Therefore, we have skipped use of the Cy5 label and have used this detection channel for simultaneous sorting of CD34^+^ cells. In our earlier work [36], we have used a lysate-based separase assay that worked with a comparable reporter peptide, but measured separase activity in protein lysates [27]. Back then, we have indeed used actin (immunostained Western blots) to normalize for separase activity. For comparing separase activity data between specimen, the use of an internal standard for normalization was crucial (i.e., actin) because the lysates from various clinical samples were derived from varying amounts of cells. Our actual assay is based on single cell measurements, and therefore, no normalization is applicable, representing the major advantage of the actual assay over the previous lysate-based separase assay. It is the key advantage of our FACS-based standard assay protocol that separase activity can be quantified on single cell level without the need of a normalization target and error-prone gel loading controls (Western blotting) (Online Resource 1).

### RNA extraction and cDNA synthesis

RNA extraction from sorted MNCs in 200 μl lysis buffer was performed using the μMACS One-step cDNA kit (Miltenyi Biotech, Bergisch Gladbach, Germany) according to the manufacturer’s instructions. cDNA synthesis was performed using the kit specific cDNA-enzyme-mix while RNA was still bound at the μMACS column. Finally, cDNA was harvested in a total volume of 70 μl elution buffer.

### *BCR-ABL1*/*GUSB* transcript detection by qRT-PCR

The LightCycler PCR and detection system (LightCycler 480, Roche Applied Science, Mannheim, Germany) was used for amplification and quantification of *BCR-ABL1* fusion gene and *GUSB* (= internal reference) transcripts. The PCR reactions were performed in 96-well plates employing a LightCycler “Fast Start DNA Master Hybridization Probes” kit (Roche Applied Science), *BCR-ABL1*- and *GUSB*-specific primers, and fluorescent probes as described by Emig and colleagues [37]. qRT-PCR for *BCR-ABL1* and *GUSB* transcripts was performed in duplicates using 15.5 μl cDNA of H- and L-fraction MNCs as template in total reaction volume of 25 μl. Detection and evaluation of *BCR-ABL1* gene expression followed the official algorithm for monitoring for residual *BCR-ABL1* transcripts [34, 38]. If no signal was detected after 45 cycles, the sample was considered *BCR-ABL1*-negative, whereas when at least one duplicate showed positivity, the whole sample was stated *BCR-ABL1*-positive. Only samples with comparable (H- vs. L-fractions) *GUSB* expression levels were evaluated. Equal numbers of cells from H- and L-fractions were analyzed for BCR-ABL1 transcript levels by qRT-PCR.

### CD34^+^ H- and L-fraction cell sorting

The flow cytometric separase activity assay was performed as previously described [35]. For CD34 staining, 5 × 10^6^ MNCs were resuspended in 1000 μl antibody binding buffer (1× PBS with 5% FKS). After centrifugation (3 min, 100×*g*), pellets were resuspended in 200 μl binding buffer, 8 μl of FcR-blocking solution (Miltenyi Biotec), and 30 μl of APC-CD34-antibody were added. The solution was incubated at 4 °C for 20 min. After the incubation step, 800 μl staining buffer were added and the solution was centrifuged for 3 min at 100×*g*. Sorting was performed as described in the section for sorting of MNCs using fluorescence (10,000 signaling events in the viable population) of Rh110 (Ex_max 488 nm_, Em_max 535 nm_) measured by FACS analysis. Cells were first gated for APC-CD34-positive cells. Measuring separase activity exclusively the separase activity–positive CD34^+^ cells were sorted in H- and L-fractions and resuspended in 150 μl StemSpanMyeloid expansion medium (StemCell Technologies, Vancouver, Canada).

### CD34^+^ cell expansion and viability assays

Expansion was performed in a 96-well plate and incubated at 37 °C and 5% CO_2_ for 3 days before proliferation/viability assays.

Viability assays were performed using the CellTiterGlow kit system (Promega, Mannheim, Germany) procedure according to the manufacturer’s instructions. Thirty microliters of cell suspension after CD34^+^ cell sorting in H- and L-fractions were mixed with 30 μl CellTiterGlow solution in a white half well microtiter plate, mixed and incubated at room temperature for 10 min. Luminescence in CD34^+^ H- and L-fractions was measured in relative luminescence units (RLU) using a plate reader (Tecan, Männedorf, Switzerland). RLU positively correlate with ATP metabolization in the cells, i.e., relative number of living cells. Ratios of H- to L-fractions were calculated.

### Assessment of replication forks by DNA fiber assays

The DNA fiber assays were performed as described previously [39–42]. In brief, 5 × 10^4^ to 5 × 10^5^ cells were seeded and incubated for 30 min with 25 μM 5-chloro-2′-deoxyuridine (CIdU) and subsequently with 250 μM 5-iodo-2′-deoxyuridine (IdU) for 60 min. Cells were diluted to a concentration of 500,000 cells/ml; therefrom, 2 μl cell suspension was spread onto SuperFrost™ slides (Fisher Scientific, Schwerte, Germany) and incubated for about 2 min. After antibody detection (rat-α-BrdU, clone BU1/75, no. MCA2060, AbD Serotec, Kidlington, UK; and mouse-α-BrdU, clone B44, no. 347580 BD Biosciences, Heidelberg Germany), fluorescence-derived microscopic images (Zeiss Axio Scope A.1, Zeiss, Jena, Germany) were analyzed using the open source ImageJ program (imagej.net) and Microsoft Excel 2016 (Microsoft, Redmond, WA, USA).

### Statistical analysis

Statistical significance of data was analyzed using the GraphPad Prism software version 6.0 (GraphPad Inc., La Jolla, USA). Values of *p* < 0.05 were considered significant. The following statistical tests were applied: Fig. 3, Kruskal-Wallis test followed by Bonferroni-Holm *p* value correction for multiple testing; Fig. 4, paired Wilcoxon signed-rank test; Fig. 5, Mann-Whitney test. Corel Draw 12 and Adobe Creative Suite 5.5 software packages were used to create pictographic artwork.

## Results

### Elevated SAD values are found in PBMNC fractions of CML patients without MMR when compared with corresponding cells of patients in MMR or deep MR and to healthy controls

**Fig. 1:**
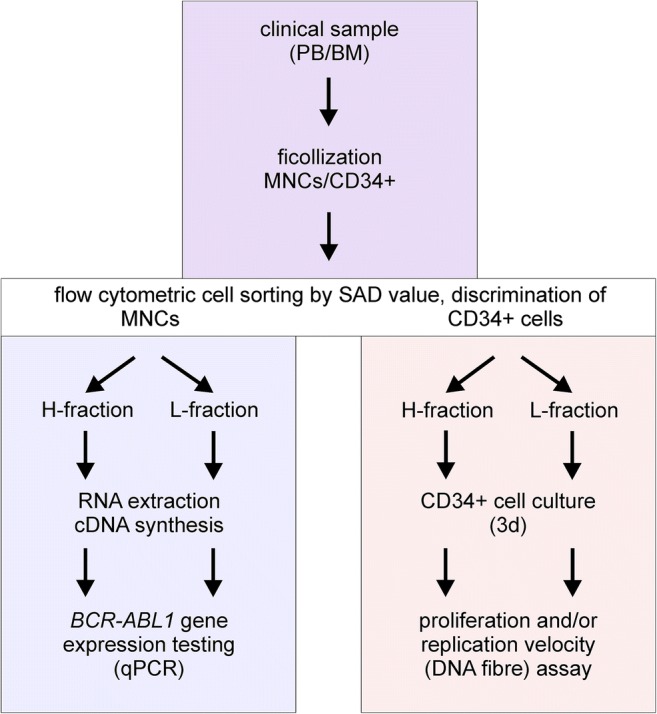
Flow chart of experimental design. After ficollization of clinical samples, the consecutive analyses were performed either on MNCs (left panel) or on CD34^+^ cells (right panel) derived from bone marrow (BM) or from peripheral blood (PB) of CML patients in chronic phase under TKI treatment. MNCs, mononuclear cells; H-fraction, MNC cell fraction with high content of separase proteolytic activity; L-fraction, MNCs with low (regular) content of separase proteolytic activity (as explained in Fig. 2); SAD value, separase activity distribution value; qRT-PCR, quantitative reverse transcriptase PCR

In order to investigate the pathophysiological context between the occurrence of hematopoietic LSC-enriched progenitor cells (CD34^+^) with elevated levels of separase activity and disease progression, we have fractionized and comparatively analyzed separase activity–positive cells derived from diagnostic samples of a total of 88 CML patients and from 14 healthy control donors. As schematically outlined in Fig. 1, fresh PB and/or BM samples were subjected to ficollization and subsequent MACS to obtain for each specimen two cell fractions in each case, one enriched for MNCs the other for CD34^+^ cells. The MNC population that makes up the majority of cells was further processed by flow cytometric cell sorting to discriminate between cells with high (H-fraction) and regular (low, L-fraction) separase activity levels and to calculate the SAD value. Both H- and L-fractions were subsequently subjected to μMACS RNA extraction, cDNA synthesis, and qRT-PCR-based *BCR-ABL1* gene expression testing. Enriched CD34^+^ cells, by far inferior in number, were processed in the same way and cultured for 3 days before cell proliferation/viability was examined. Whenever an adequate number of cells were available of H- and L-fractions after proliferation/viability testing, we measured replication fork velocity by DNA fiber assays [40, 41].

It has to be emphasized that due to limitations in the available amounts of diagnostic material and due to the fact that fresh and unfrozen diagnostic material had to be used for unbiased separase activity testing results, not all types of analyses could be performed with each patient sample. Moreover, it is to note that only a minor fraction (3 to 8%) of the analyzed CD34^+^ BM cells was mitotically active (i.e., separase activity–positive), and therefore, as many BM cells as possible (available) had to be subjected to flow cytometric analysis according to our standardized protocol [35] to obtain at least approximately 50–500 cells in CD34^+^ H-fractions.

**Fig. 2:**
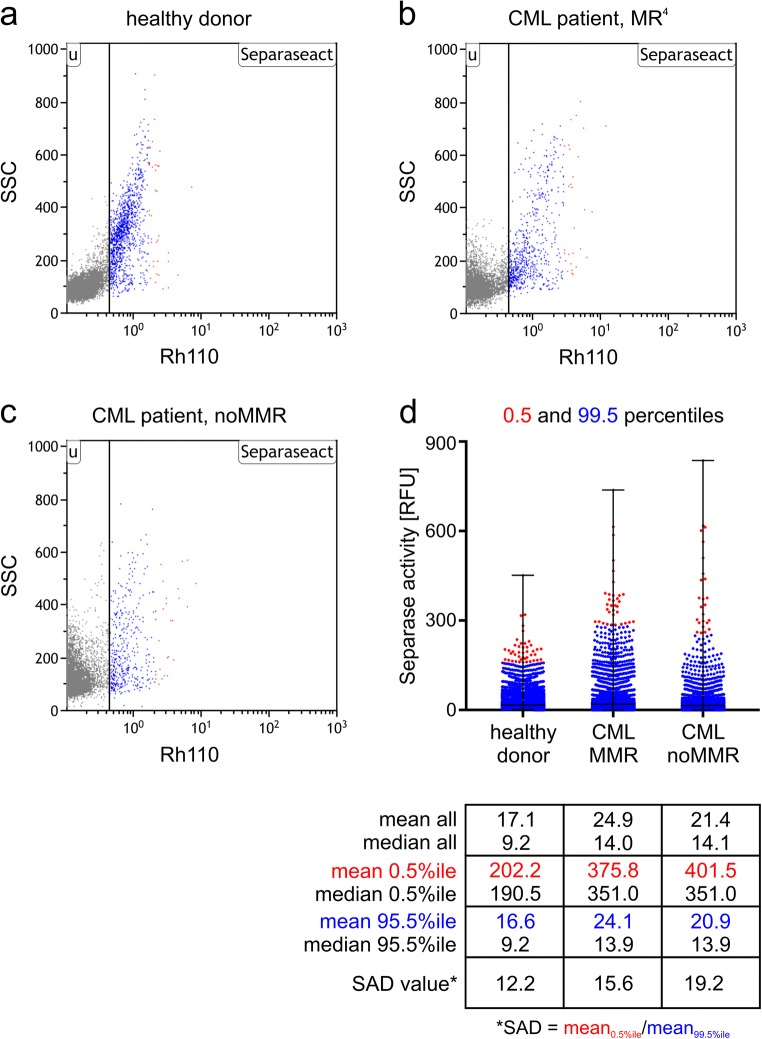
Showcase visualization of separase proteolytic activity measurement on single cell level and calculation of SAD values. For representative illustration of SAD value calculation fresh PBMNCs derived from a single healthy donor (**a**) and from two CML patients, one from the MMR cohort (**b**) and one from the noMMR cohort (**c**) were analyzed by the flow cytometry–based separase activity assay. Flow cytometry data is depicted as dot blot representation (scatter blot). Separase activity–positive cells are shown in color (blue and red). In **d**, a corresponding flow cytometry event-derived dot blot represents separase-active cells of **a**, **b**, and **c** ordered by their Rh110 fluorescence intensities (= intracellular separase activity). The relative distribution of Rh110 intensities has been accentuated by coloring cells above the 99.5 percentile (= 0.5% of separase-positive cells) in red and cells below the 99.5 percentile (= 99.5% of separase-positive cells) in blue. The quotient of mean Rh110 fluorescence intensities (*M*_0.5%_/*M*_99.5%_) was calculated to serve as numerical value of intercellular separase activity distribution among cells in the three samples. The table below gives the mean and median for all, for the 0.5 percentile and the 99.5 percentile of cells, as well as the resulting SAD values. SSC, sideward scatter; Rh110, rhodamin 110 fluorescence; MMR, major molecular response; MR^4^, molecular response with a ratio *BCR-ABL1/ABL1* log reduction of 4 under TKI therapy; SAD value, separase activity distribution value

The FACS assay allows generation of separase activity profiles that not only tell about the number of separase-positive cells within a sample but also about the range of intercellular variation in separase activity levels within the cell fraction of interest. As shown in Fig. 2, the assay can be used to comparatively quantify the separase proteolytic activity in MNCs of healthy (a) and CML specimen (b, c). Furthermore, measuring fluorescence on single cell level allows calculation of the SAD value that serves as a numerical measure of intercellular separase activity distribution among single cells in the analyzed sample (Fig. 2d). In other words, the SAD value is a calculative value for the occurrence of cells with prominent separase activity even though the number of these cells may be low as demonstrated previously [35, 43]. Thus, analysis of the healthy donor, CML patient in MR^4^, and CML patient without MMR revealed SAD values of 12.2, 15.6, and 19.2, respectively (Fig. 2d). So the highest SAD value was obtained for the noMMR patient.

It is generally accepted that tumors originate from single aberrant cells (clonality of tumors) and the subsequent clonal evolution of tumors also depends on the emerging of single cells with additional genetic mutations that may attain proliferative advantage and/or resistance, thereby avoiding therapeutic pressure [44]. Therefore, the detection and molecular characterization of single cells with extraordinary features are of special interest in terms of elucidating disease-relevant phenotypes concurring with impaired differentiation and increased proliferation.

Here, while analyzing the separase proteolytic activity in circulating MNCs/CD34^+^ cells in patients with CML under TKI treatment, we observed the occurrence of a small number of cells with conspicuously enhanced levels of separase activity. From a statistical view, these may simply be termed as “outliers,” but not so in the tumorbiological sense, where these cells may drive tumor heterogeneity and clonal evolution under therapy. Therefore, when arithmetically describing the occurrence of such cells in our sample cohorts by the previously established SAD value (ratio of means 0.5% percentile/99.5% percentile), we chose for calculation the mean instead of the median, because the latter does not take into account the small cell population with conspicuously enhanced levels of separase activity. This is shown in the table accompanying Fig. 2d where 0.5% and 99.5% percentiles, mean and median values, and the resulting SAD values are exemplarily shown for one healthy donor, one CML patient with MMR and one patient not having achieved MMR (noMMR). Using the median for SAD value calculation, no differences between MMR and noMMR patient would be detected. Therefore, the use of mean is reasonable when the influence of a few single cells with extraordinary separase activity is assessed.

**Fig. 3:**
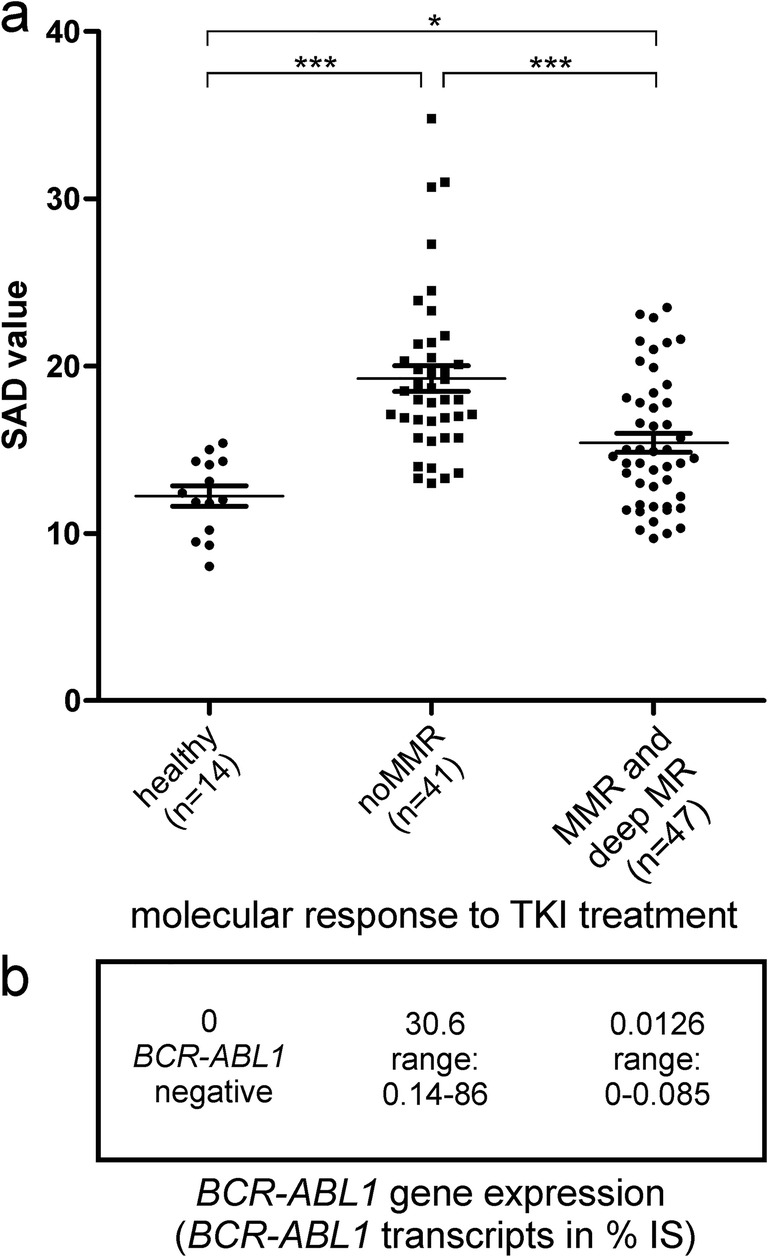
Comparison of separase activity distribution (SAD value) in vital separase activity–positive PBMNC preparations. **a** The SAD values of healthy donors (*n* = 14) were compared with SAD values of patients without major molecular response (noMMR, *n* = 41, including samples from the time point of initial diagnosis and after relapse) and to SAD values of CML patients that have reached major molecular response (MMR) or deep molecular response (MR, including MR^4^, MR^4.5^, or MR^5^) under continuous TKI maintenance therapy at time of sampling (*n* = 47). **b** Mean and range of *BCR-ABL1* transcripts in % according to the international standard (IS) as measured in the tested clinical sample by routine CML *BCR-ABL1* monitoring (qRT-PCR). For abbreviations, see legend of Fig. 2. Statistical analysis: Kruskal-Wallis test followed by Bonferroni-Holm *p* value correction for multiple testing

As depicted in Fig. 3a, measurement of separase activity and comparison of calculated SAD values between healthy controls (*n* = 14), CML patients without MMR (*n* = 41), and patients in MMR or deeper (*n* = 47) revealed the highest SAD values in the cohort of patients with the highest *BCR-ABL1* expression (noMMR, mean 19.2 ± 4.38, range 13.0 to 34.8) when compared with the control group (healthy, mean 12.2 ± 2.22, range 8.0 to 15.4; 95% CI, − 10.16 to − 3.868) and the therapy responder group (MMR or deeper, mean 15.4 ± 3.86, range 9.7 to 23.5; 95% CI, 1.663 to 6.010). The positive correlation of high SAD values and high *BCR-ABL1* gene expression levels (see Fig. 3b) suggests that the SAD value derived from granulocyte-depleted MNC fractions may positively correlate with disease status/progression in CML.

### CD34^+^ cell fractions with high separase activity levels (H-fractions) are associated with enhanced proliferation and BCR-ABL1 gene expression

**Fig. 4:**
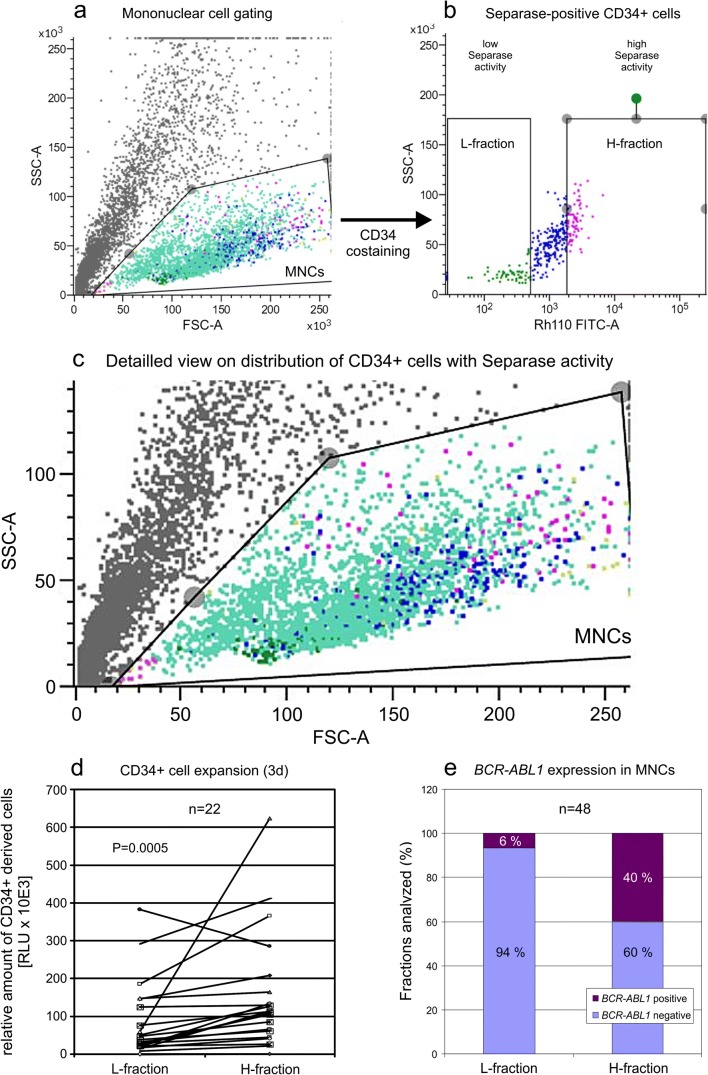
Comparative analysis of H- and L-fractions derived from CD34^+^ cells enriched by magnet-assisted cell sorting (MACS) from CML clinical specimen. **a** Flow cytometric scatter plot showing the localization of separase- and CD34-positive H- and L-fractions within the total MNC population of a *BCR-ABL1*-positive BM sample. **b** Gating strategy for sorting separase-active CD34^+^ cells into fractions with high (H-fraction) and low (regular, L-fraction) separase proteolytic activity. Cutoffs were established empirically based on the borders between 0.5 and 99.5 percentiles as depicted in Fig. 2d. **c** Magnification of **a** showing a detailed view on distribution of CD34^+^ cells with separase activity within the MNC gate. While L-fraction cells cluster due to low granularity and small size, cells of the H-fraction are more widely scattered with a tendency to high granularity and bigger size. H-fraction cells are depicted in magenta; the L-fraction cells are given in dark green. **d** Proliferation/viability analysis of H- and L-fractions by ATP quantification employing CellTiter-Glo luminescent cell viability assay after 3 days of propagation of 10,000 cells each in StemSpan Serum-Free Expansion Medium II. Statistical analysis: paired Wilcoxon signed-rank test, *p* = 0.0011. **e** Qualitative assessment of *BCR-ABL1* gene expression testing in MNC-derived H- and L-fractions by qPCR after microscale total RNA extraction and cDNA synthesis (*n* = 48). Equal numbers of cells from H- and L-fractions were analyzed for BCR-ABL1 transcript levels by qRT-PCR. SSC-A, side scatter area; FSC-A, forward scatter area; Rh110 FITC-A; spectral channel for measurement Rh110 fluorescence (= separase proteolytic activity); MNCs, mononuclear cells; RLU, relative luminescence units; CML, chronic myeloid leukemia

In order to further investigate in more detail the cells that are in charge of the elevated SAD values observed in the “noMMR” cohort, we have exclusively sorted and analyzed separase-positive CD34^+^ cells into H- and L-fractions. Figure 4 a and b show a representative sorting experiment where CD34^+^ cells were enriched from the PB sample of a relapsed CML patient (55% BCR-ABL1 transcripts according to IS). Figure 4 c is a magnification of Fig. 4a and gives a detailed view on distribution of CD34^+^ cells with separase activity within the corresponding MNC gate. While L-fraction cells cluster due to low granularity and small size, cells of the H-fraction are widely scattered with a tendency to high granularity and bigger size. H-fraction cells are depicted in magenta; the L-fraction cells are in dark green. These findings suggest that cells of the H-fraction may contain more cytoplasm and may therefore differ also metabolically from L-fraction cells.

To test for viability and proliferative capability, equal numbers of cells (*n* = 150 to 2000, depending on clinical sample size) derived from H- and L-fractions (the H-fraction always limiting) were propagated in expansion medium followed by cell viability assay. As shown in Fig. 4d, an increase in luminescence (mean 3.3-fold, range 0.74 to 11.4; *p* = 0.0011 in Wilcoxon signed-rank test) was detected in 21 H-fractions of 22 clinical specimen when compared with the corresponding L-fraction of the same patient sample. Concomitant manual microscopic counting of CD34-derived colonies/cells confirmed that H-fractions feature CD34^+^ cells with higher proliferative performance than the L-fractions.

In order to investigate additional factors that may contribute to the proliferative advantage found in cells of the H-fraction, we tested H- and L-fractions for *BCR-ABL1* gene expression. Due to limited cell numbers available from clinical diagnostic, PB/BM samples ficollized MNC preparation instead of CD34^+^ fractions was used for qRT-PCR experiments. This enabled collection of sufficient cells (*n* = 5000 to 10,000 cells) for RNA extraction and analysis of *BCR-ABL1* gene expression analysis in H- and L-fractions.

Despite the high sensitivity of qRT-PCR, only *BCR-ABL1* transcript numbers of up to 15 copies were measured in the *BCR-ABL1*-positive H- and L-fractions indicating very low *BCR-ABL1* gene expression levels near the technical detection limit (LOD_95%_ = 3 molecules/assay, [45]. Therefore, we chose qualitative over quantitative evaluation of the *BCR-ABL1* expression data as shown in Fig. 4e. The analysis of 48 paired clinical specimen revealed predominant *BCR-ABL1* gene expression activity (transcript positivity) in H-fractions (40%) whereas only in 6% of the corresponding L-fractions *BCR-ABL1* transcripts could be detected. While in 4 samples with high SAD value both H- and L-fractions were tested *BCR-ABL1*-positive, no *BCR-ABL1* positivity could be detected in fractions of samples with low SAD values (MMR or deeper) that display less than 0.1% *BCR-ABL1* transcripts (according to IS). Overall, our data point to an association between *BCR-ABL1* gene expression and proliferative capacity in hematopoietic cell fractions with elevated/prominent separase proteolytic activity of CML patients without MMR (initial diagnosis) or after relapse.

**Fig. 5:**
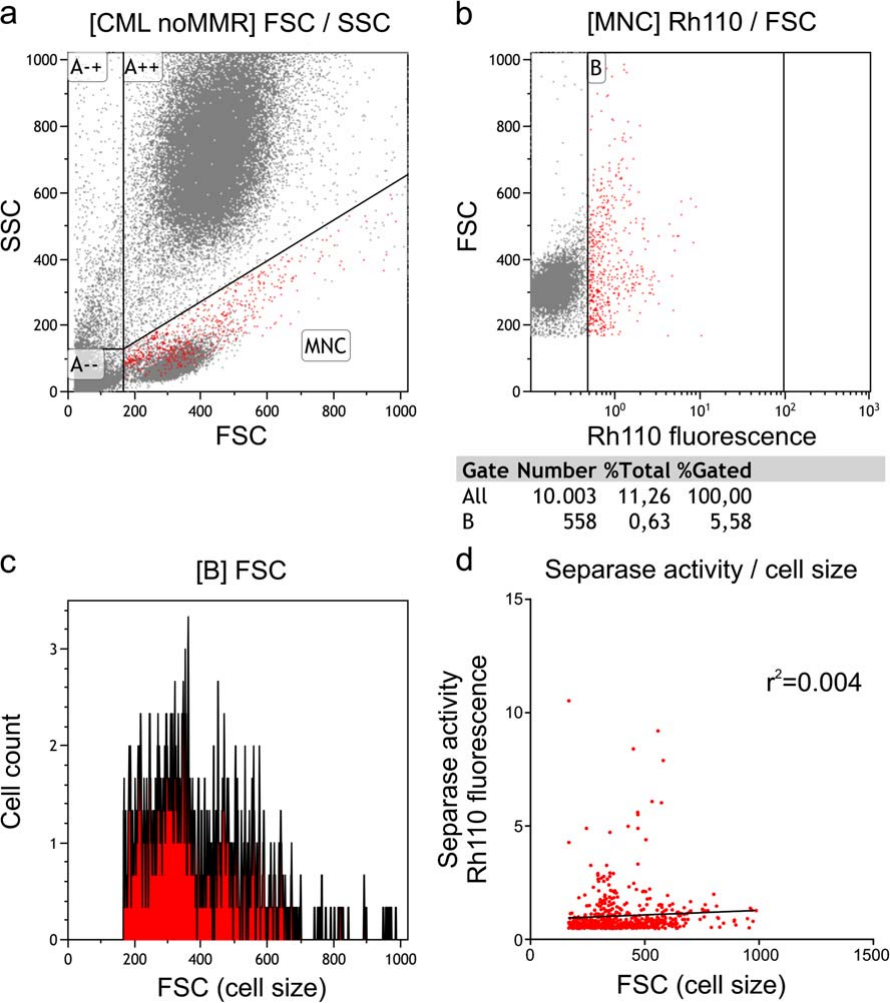
Correlative analysis of separase proteolytic activity and cell size using MNCs of a CML patient (noMMR). **a** Gating of ficolized blood cells using sideward scatter (SSC) as a measure of the granularity of the cells and forward scatter (FSC) as a measure of cell size. **b** Size distribution of separase-active MNCs (gate B) given by released Rh110 fluorescence upon peptidic substrate cleavage. **c** Histogram showing correlation of separase-positive cell counts with cell size reveals normal distribution of values. **d** Calculation of the correlation coefficient (*r*^*2*^ = 0.004) confirms lack of correlation between cell size and signal strength in separase activity assaying

One could argue that the observations of L- and H-fraction cells showing different separase proteolytic activity is solely dependent on cell size, because peptidic substrate uptake may be a function of cell size and therefore may be limiting the assay efficacy in small cells. This can be clearly excluded. Figure 5 showing representative cell sorting data from one patient with noMMR indicates that the Rhodamine uptake or cleavage is independent of cell size since the measured separase activity (released RH110 fluorescence) does not correlate with cell size. In Fig. 5a, the sorting of the cells by size and granularity is shown. For all cells identified as MNCs, the cell size was compared with separase activity (Fig. 5b), revealing no correlation. One of the small cells is, e.g., the one for which the highest separase activity was measured. For the separase-positive cells as depicted in Fig. 5b, the cell size was related to the cell number (Fig. 5c), revealing a normal distribution pattern. In addition, calculation of the correlation coefficient (*r*^2^ = 0.004) confirmed lack of correlation between cell size and fluorescence signal strength as measured by our separase assay (Fig. 5d). Therefore, we rule out technical limitations compromising the obtained separase activity data measured in our clinical samples.

To further exclude the possibility that limited substrate saturation may compromise the separase activity results, we clearly show in time kinetic experiments on human MNCs (compare Online Resource 1) that substrate saturation is achieved under standard assay conditions (90-min incubation time, 10 μM peptide) in an incubation time between 60 and 120 min despite of continuous cleavage by active separase. Therefore, we consider the substrate not limiting and the obtained signals are only due to separase activity in the applied time window of incubation.

### CD34^+^ cells with high separase activity levels (H-fractions) and increased proliferation rates display altered replication fork dynamics (decreased replication fork speed)

**Fig. 6:**
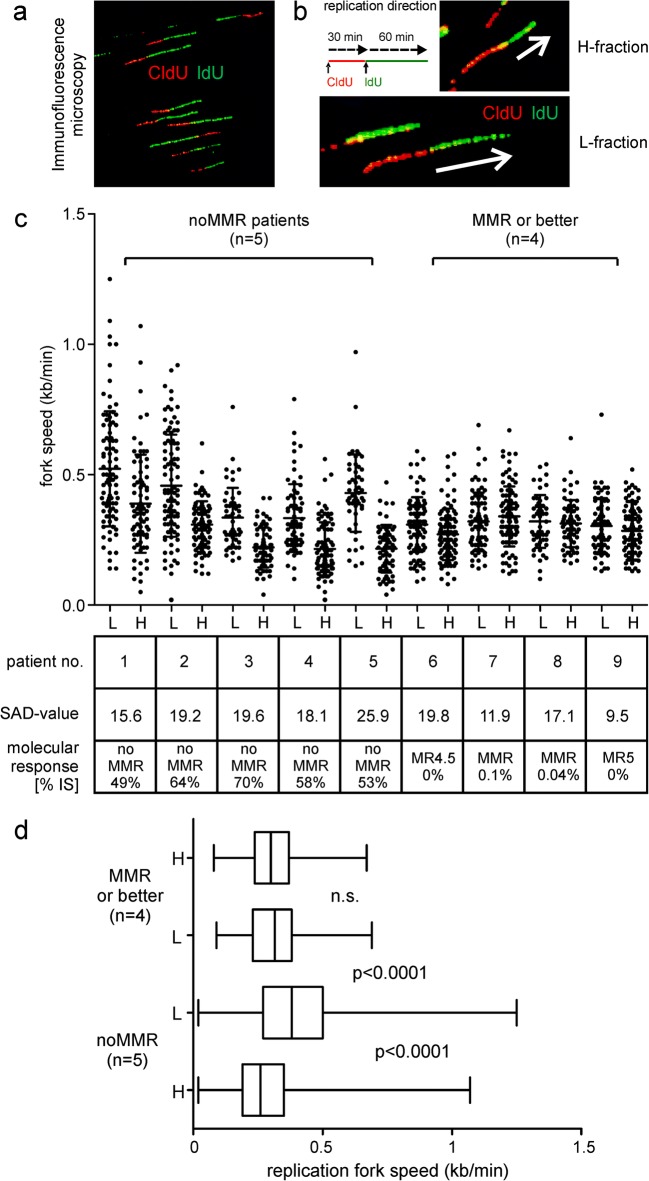
Assessment of replication fork speed in CD34^+^ cells of H- and L-fractions by chromosomal fiber fluorography. **a** Visualization of replication fork dynamics in separase activity–positive cells by immunofluorescence microscopy. **b** Representative images of genomic DNA molecules analyzed by combing. White arrows exemplify unidirectional replication events of varying velocity. For single-molecule analysis, cells were labeled with CldU for 30 min (first pulse, red), then washed and pulsed with IdU for further 60 min (second pulse, green). **c** Measurement of CldU and IdU incorporation in CD34^+^ cells of sorted L- and H-fraction from “noMMR” patients (*n* = 5) and “MMR or better” patients (*n* = 4). For each sample pair, SAD value and molecular response (*BCR-ABL1* quotient IS) are shown. **d** For comparative analysis of fork velocities, at least 75 tracks of unidirectional forks were measured per sample as given by the box plots for noMMR patients (*n* = 5) and for patients in MMR or better (*n* = 4). Statistical analysis: Mann-Whitney test. CIdU, 5-chloro-2′-deoxyuridine; IdU, 5-iodo-2′-deoxyuridine; L/H fraction, CD34^+^ cell fractions with low (regular)/high (increased) content of separase proteolytic activity; SAD value, separase activity distribution value; MMR, major molecular response; MR^4^ indicates ≥ 4-log reduction (*BCR-ABL* IS ≤ 0.01%), MR^4.5^ indicates ≥ 4.5-log reduction (*BCR-ABL* IS ≤ 0.0032%), and MR^5^ indicates ≥ 5-log reduction (BCR-ABL IS ≤ 0.001%) of measurable *BCR-ABL1* transcripts by qRT-PCR

Since separase cleaves cohesin complexes that hold sister-chromatids together and has been reported to be involved in controlling replication fork dynamics and higher order chromatin architecture, we set out to investigate the replication fork speed in H- and L-fractions by the DNA fiber technique [40, 41]. Replication fork speed may directly influence proliferation and cell doubling time but may also contribute to genomic instability via replication stress [42] and to altered interphase DNA repair [22], both phenomena potentially contributing to disease progression in CML [46]. We have performed pulse chase CIdD/IdU incorporation experiments on genomic DNA of H- and L-fractionized cells of 9 clinical CML specimen (Fig. 6a, b). Five samples (patient no. 1–5) resemble CD34^+^ cells of patients at initial diagnosis (untreated, noMMR); four samples (patient no. 6–9) are derived from BM of patients that have achieved MMR or better under TKI treatment, and therefore, isolation of CD34^+^ cells from PB samples was not successful/possible. For all specimen replication, fork velocities for H- and L-fraction cells were calculated according to published algorithms (Fig. 6c) [40, 41].

As depicted in Fig. 6d, the replication fork velocity in L-fractions of noMMR patients was increased when compared with the corresponding H-fractions (median: 0.38 vs. 0.26, *p* < 0.0001) and to L- and H-fractions of patients with MMR or better (*p* < 0.0001), the latter showing no differences between L- and corresponding H-fractions (n.s.). This observation concurred with the observed SAD values (noMMR: mean 19.6, range 15.5 to 25.9 vs. MMR or better: mean 14.6, range 9.5 to 19.8) and MR data (*BCR-ABL1* quotient IS of noMMR: mean 59%, range 49 to 70% vs. MMR or better: mean 0.04%, range 0 to 0.1%) as given in Fig. 6c lower panel. Our data suggest that CD34^+^ cells from noMMR patients with increased separase activity (H-fractions) show a 0.68-fold decreased replication fork speed when compared with CD34+ cells with regular separase activity levels.

## Discussion

We found that diagnostic PB samples from TKI-treated noMMR patients that in general contain only 3 to 8% separase activity–positive cells comprise a small number of cells with conspicuously high levels of separase activity resulting in high SAD values [35]. These cells range outside the Gaussian distribution for intracellular fluorescence that was measured in separase-active cells of samples derived from CML patients in remission or from healthy donors (= low SAD values). This small number of cells with exceedingly high separase activity (50 to 500 cells per sample) was enriched by flow cytometric cell sorting (H-fraction) and was object of our investigations.

Comparative analysis of H-fractions revealed preferential positivity for *BCR-ABL1* gene expression when compared with corresponding cells of L-fractions (noMMR). Regarding the low number of cells collectable in H-fractions and the fact that the majority of *BCR-ABL1* transcript level measurements ranged near the detection limit (LOD_95%_ = 3 transcripts), one might assume that only a small number of separase-active cells (H-fraction) are Ph+ as well. However, the observed *BCR-ABL1* transcript numbers do not allow to deduce Ph+ cell counts within H-fractions. TKI treatment is known to reduce the proliferative turnover of LSCs. These quiescent CML LSC–enriched progenitor cells express very low levels of *BCR-ABL1* concurring with less sensitivity to TKIs and *BCR-ABL1*-independent persistence [11, 13]. It cannot be excluded that LSCs may count among the CD34^+^ cells with elevated separase activity as these cells displayed enhanced proliferation/viability and a decreased replication fork velocity that concurs with an altered morphology when compared with corresponding cells of L-fractions. Granularity (SSC-A) and size (FSC-A) of the latter were clearly outperformed by cells of the H-fraction suggesting that cells of the H-fraction are bigger and therefore may be metabolically more active/proliferative than cells of the L-fraction.

**Fig. 7:**
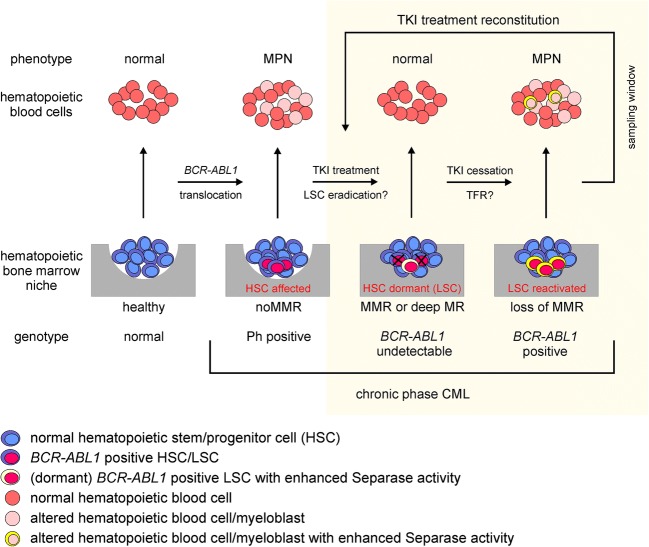
Hypothetic model illustrating the occurrence and the role of HSC/LSC with enhanced separase activity in the hematopoietic bone marrow niche of patients with CML for disease progression under TKI treatment and after TKI treatment cessation. For explanation, see “Discussion.” The yellow box indicates the origin of clinical samples used in this survey. ACA, additional chromosomal alterations; CML-CP, CML in chronic phase; CML-AP/BP, CML in accelerated phase/blastic phase; TKI, tyrosine kinase inhibitor; HSC, hematopoietic stem cell; LSC, leukemic stem cell; Ph Philadelphia chromosome; MR, molecular response; TFR, treatment-free remission

Since dysregulated separase is well-known as an oncogene and a driver of genomic instability (aneuploidy), we establish here a model hypothesis (Fig. 7) on how elevated activity of separase may be induced in *BCR-ABL1*-positive cells and how an overactive separase albeit detectable only in a limited number of disease-related hematopoietic LSC-enriched progenitor cells may potentially promote long-term survival and reactivation/clonal evolution of dormant LSC concurring with loss of MMR after TKI treatment cessation.

As sketched in Fig. 7, the *BCR-ABL1* gene translocation resulting in a constitutively active BCR-ABL1 TK renders normal hematopoiesis incipiently into a moderate myeloproliferative neoplasm (CML-CP) that is characterized by increased white blood cell counts including appearance of more or less undifferentiated *BCR-ABL1*-positive myeloid precursors in the PB of patients. Although stringent TKI treatment can reduce tumor burden (as routinely measured in number of *BCR-ABL1* transcripts in PB and/or BM) down to undetectable levels (= deep MR), it appeared that current TKIs are unable to eradicate LSCs that probably represent a small number of persisting hematopoietic pluripotent stem cells within the hematopoietic BM niche [47]. LSCs remain in dormant phase, show *BCR-ABL1*-independent survival, and are resistant to apoptosis [13, 48]. It is assumed that TKI treatment reduces turnover and self-renewal of *BCR-ABL1*-positive LSCs but also facilitates a “low mutator” phenotype. Namely, LSCs neither exhibit mutation-driven TKI resistance nor progress to advanced phase but keep acquiring increased levels of radical oxygen species (ROS) and are supposed to activate other mechanisms preparing the ground for later clonal evolution and tumor progression [11, 12].

Although the clinical efficacy of TKI treatment in achieving deep MR is excellent, there was a recent proof that as a side effect of TKI administration, a posttranslational activation of separase may occur as demonstrated in a number of in vitro experiments comparatively performed on imatinib-treated *BCR-ABL1*-positive and *BCR-ABL1*-negative cell lines [36]. In previous work, we found that the posttranslationally stimulated separase proteolytic activity under imatinib treatment rendered b3a2 fusion type-related CML cells more prone to aneuploidy and clonal evolution, thereby confirming cytogenetic findings within a cohort of 1151 Philadelphia chromosome-positive chronic phase patients of the randomized CML-study IV that were examined on the incidence of newly arising additional chromosomal alterations (ACAs) under prolonged imatinib treatment. This points to an influence of high separase activity for the induction of gross genomic mutations in CML cells [30].

Since separase overactivity has been repeatedly reported to be associated with the occurrence of numeric centrosome aberrations and aneuploidy, the increased separase activity levels observed in CD34^+^ cells (H-fractions) may represent a novel cardinal feature of LSCs [43, 49]. This mechanism of genomic destabilization may pave the way for residual tumor cells to clonal evolution and a more efficient therapy for survival. As a novel “mutator” mechanism, unscheduled proteolysis of intracellular targets by an “overactive separase” may join the list of so far known tumor escape strategies such as upregulated PI3/AKT pathway, AKT-mediated phosphorylation of FOXO transcription factors, altered Hedgehog and Wnt pathways, and constitutive activation of JAK/STAT signaling [11]. Thus, this demonstrates that TKI treatment itself may exert adverse effects that give rise to *BCR-ABL1*-independent pathways for promoting tumor cell survival and evolution of the malignant clone. Moreover, it points to the LSC as a potent source of clinically relevant mutations suggesting that CML is constantly evolving at a molecular level even in CP and under stringent TKI therapy [5].

The observation of reduced replication fork velocity in CD34^+^ of MMR-derived cells with low levels of separase activity when comparing MMR to noMMR cohorts may reflect the dormance and/or reduced self-renewal of the affected CD34^+^ cells. This is in line with experiments of Cucco and co-workers who reported on the role of separase for genomic instability in HeLa (cancer cells) and NHDF (normal human dermal fibroblasts) cells. They found unexpectedly that the depletion of separase increased the fork velocity about 1.5× and led to altered checkpoint responses, thereby providing evidence that separase participates in genomic stability maintenance by controlling replication fork speed [21]. Conversely, we state in accordance with the findings of Cucco that an increased separase activity obviously decreases the fork velocity (noMMR H- vs. L-fractions). The impact of this effect on proliferation/survival of LSCs that is most likely indicative for replication stress is still an open question [42].

Assuming that high continuous separase activity levels in residual dormant LSCs (= the “mutator phenotype”) may affect their long-term genomic stability and support their survival under continuous TKI treatment (Fig. 7), cessation of TKI treatment as conducted within TKI stopping trials may immediately affect TFR by LSC reactivation. At worst, loss of deep MR or MMR will make TKI treatment reconstitution necessary within a short period of time as found in recent stopping trials [50–52]. It is currently unclear whether and how high separase activity levels may contribute to reactivation of quiescent LSCs. Nevertheless, cells with high separase activity levels, potential descendants of reactivated LSCs, will also appear in the PB as we detected such cells in the H-fractions derived from PBMNC preparations of noMMR/relapse patients (compare Fig. 7, state “LSC reactivated”). It is conceivable that the CD34^+^ LSCs with high separase activity levels themselves may appear in the PB as it has been described that HSC spontaneously leave their niches daily and egress in circulation without entering into cell cycle. In this case, a specific type of niche was not absolutely required to keep LSCs quiescent as they can stay in G0 phase of cell cycle in circulation [53].

In conclusion, the represented data suggests that the loss/lack of achievement of MMR in TKI-treated CP CML coincides with the occurrence of circulating CD34^+^ progenitor cells with elevated separase activity. For these cells, enriched fractions showed preferential *BCR-ABL1* gene expression positivity, enhanced proliferation/viability in vitro, and decreased replication fork velocity. We propose a hypothetical model suggesting that enhanced separase proteolytic activity may represent a novel cardinal feature of the leukemic clone supporting the “mutator” phenotype of quiescent LSCs under long-term TKI treatment. This novel mechanism of genomic destabilization may pave the way for residual tumor cells to clonal evolution and a more efficient therapy survival. In this context, it would be of high interest to better delineate the mechanisms of the observed high separase activity. This will be the focus of future studies. Moreover, evaluating patients of different phases of the treatment journey would be desirable as well but is currently not feasible due to the lack of corresponding longitudinal samples including chronic, accelerated, and blastic phase specimen from the same patient. However, measurement of separase activity may be useful as surrogate marker of rampant proliferative capacity for prediction or monitoring of CML molecular response before and after TKI treatment cessation.

## Electronic supplementary material

ESM 1 (DOCX 614 kb)

## Data Availability

All generated data are available within the manuscript.
