# Supplementary material for: Separase activity distribution can be a marker of major molecular response and proliferation of CD34+ cells in TKI-treated chronic myeloid leukemia patients
Source: Ann Hematol. 2020 Apr 6;99(5):991–1006. doi: 10.1007/s00277-020-04007-4 (PMC7196950; doi:10.1007/s00277-020-04007-4)
Supplement: Supplementary file 1 — (DOCX 614 kb) [file 277_2020_4007_MOESM1_ESM.docx]

**Online Resource 1** accompanying **AOHE-D-19-00899R1**

Journal: „Annals of Hematology“

Title: Separase activity distribution can be a marker of major molecular response and proliferation of CD34+ cells in TKI-treated chronic myeloid leukemia patients

Autors: Birgit Spiess*^1^, Helga Kleiner^1^, Johanna Flach^1^, Alice Fabarius^1^, Susanne Saussele^1^,

Wolf-Karsten Hofmann^1^, Wolfgang Seifarth^1^

Affiliation: ^1^ Department of Hematology and Oncology, University Hospital Mannheim, Medical Faculty Mannheim of the Heidelberg University

* **Correspondence:**

PD Dr. sc. hum. Birgit Spiess

Universitätsklinikum Mannheim GmbH

III. Medizinische Klinik

Hämatologie und Onkologie

Wissenschaftliches Labor

Pettenkoferstraße 22

D-68169 Mannheim

Telefon: +49-621-383-71301

Telefax: +49-621-383-71329

E-Mail: [birgit.spiess@medma.uni-heidelberg.de](mailto:birgit.spiess@medma.uni-heidelberg.de)

**Substrate saturation/normalization in Separase activity assays**


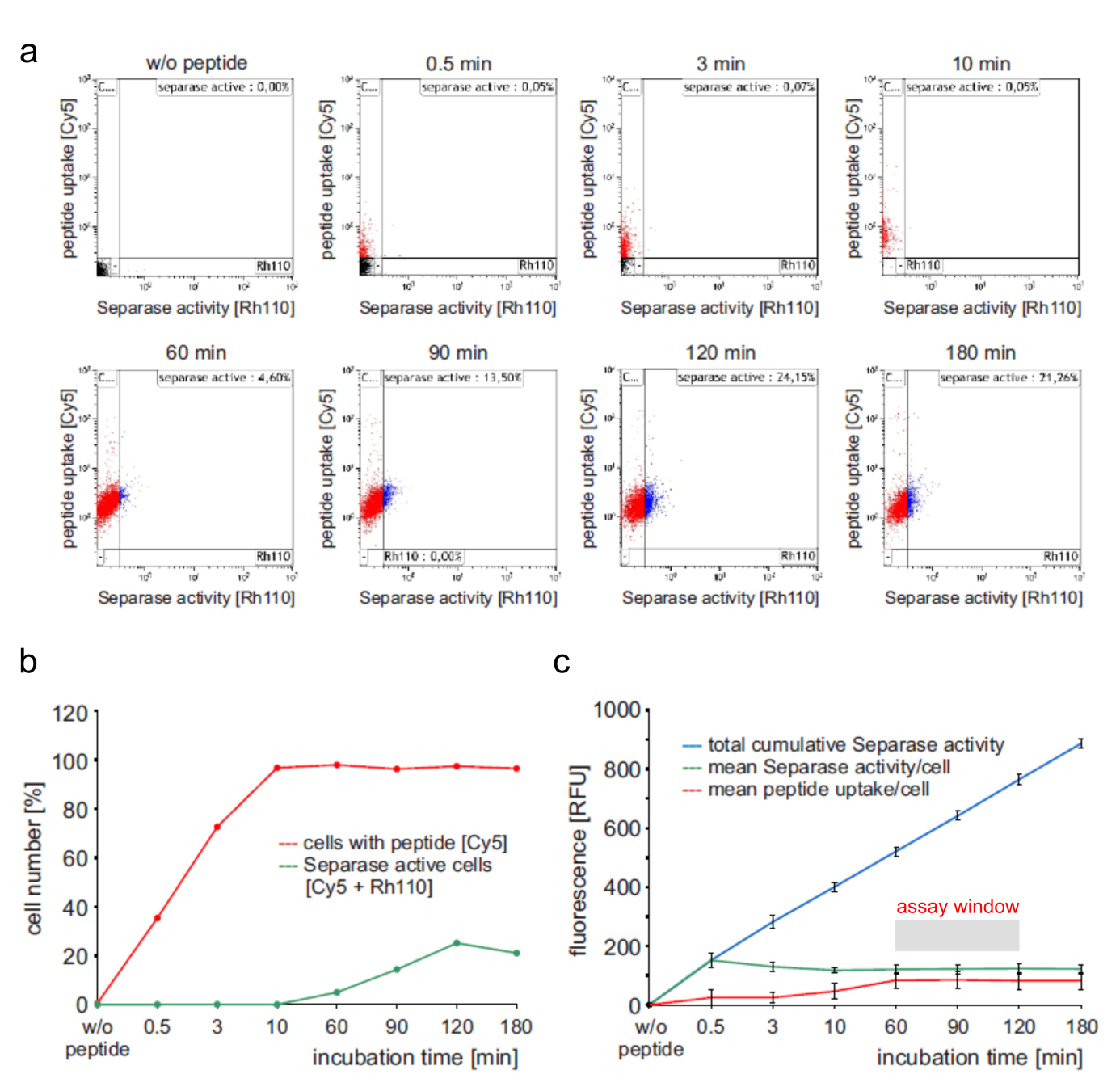


Supplementary Figure 1: **Influx/uptake kinetics of Separase activity assay-related reporter peptide in living human peripheral blood MNCs derived from of a healthy donor**. A dual-labelled reporter peptide (Cy5- and rhodamine 110 (Rh110)-biconjugated Rad21 cleavage site peptide ([Cy5-D-R-E-I-M-R]2-Rh110) as smart probe) was used to test the kinetics of substrate uptake and signal generation at 10µM substrate concentration for varying incubation times at 37°C reaction temperature. **a** Flowcytometric scatter plots for eight time points (t=0 and 30s to 180min). Count events in red depict cells positive for substrate uptake (Cy5), whereas events in blue represent cells with internal Separase activity that have already cleaved reporter peptide molecules (release of Rh110 fluorescence). It should be noted that incremental cleavage of the reporter peptide (movement to the right on the x-axis) does not deplete the intracellular concentration of the fluorogeneous peptidic substrate as the red signal events stay at the same y-axis location within the scatter plots. This proves that uncleaved reporter peptide is always available in molar excess and not limiting the Separase activity assay. **b** Diagram based on data of **a** showing that after 10min incubation all cells are Cy5 positive whereas accumulation of Rh110-positive cells reaches its maximum at the 120min timepoint. **c** Diagram based on data of **a** showing mean values of Cy5 and Rh110 fluorescence per cell indicating that a steady equilibrium for both substrate uptake and signal forming/retention has formed after 60 min of assay incubation time. Therefore, we considered a 90min incubation time (10µM substrate concentration, 37°C reaction temperature) appropriate as standardized assay conditions to reliably assess Separase proteolytic activity in clinical samples of interest. It should be emphasized that due to the steady state equilibrium and the single cell-based nature of the Separase assay no internal normalization is necessary. This is in clear contrast to lysate-based Separase assays that work with a comparable reporter peptide, but measure Separase activity in protein lysates. Here, when comparing Separase activity data between clinical specimen the use of an internal standard for normalization is crucial (i.e. actin) because the lysates from various clinical samples are derived from varying amounts of cells. It is the key advantage of our FACS-based standard assay protocol, that Separase activity can be quantified on single cell level without the need of a normalization target and error-prone gel loading controls (Western blot immunostaining experiments).
